# COVID-19—A Trigger Factor for Severe Immune-Mediated Thrombocytopenia in Active Rheumatoid Arthritis

**DOI:** 10.3390/life12010077

**Published:** 2022-01-06

**Authors:** Anca Bobircă, Florin Bobircă, Ioan Ancuța, Anca Florescu, Mihai Bojincă, Alice Muscă, Dan Nicolae Florescu, Lucian Mihai Florescu, Romina Marina Sima, Alesandra Florescu, Anca Emanuela Mușetescu

**Affiliations:** 1 Department of Internal Medicine and Rheumatology, Carol Davila University of Medicine and Pharmacy, 050474 Bucharest, Romania; anca.bobirca@umfcd.ro (A.B.); ioan.ancuta@umfcd.ro (I.A.); mihai.bojinca@umfcd.ro (M.B.); 2 Department of Internal Medicine and Rheumatology, Dr I. Cantacuzino Clinical Hospital, 011437 Bucharest, Romania; anca-teodora.florescu@rez.umfcd.ro (A.F.); alice_musca@yahoo.com (A.M.); 3 Department of General Surgery, Carol Davila University of Medicine and Pharmacy, Dr I. Cantacuzino Clinical Hospital, 050474 Bucharest, Romania; florin.bobirca@umfcd.ro; 4 Department of Gastroenterology, University of Medicine and Pharmacy of Craiova, 200349 Craiova, Romania; dan.florescu@umfcv.ro; 5 Department of Radiology and Medical Imaging, University of Medicine and Pharmacy of Craiova, 200349 Craiova, Romania; lucian.florescu@umfcv.ro; 6 Department of Obstetrics and Gynecology, “Bucur” Maternity, “Saint John” Clinical Emergency Hospital, 077160 Bucharest, Romania; romina.sima@umfcd.ro; 7 Department of Rheumatology, University of Medicine and Pharmacy of Craiova, 200349 Craiova, Romania; anca.musetescu@umfcv.ro

**Keywords:** thrombocytopenia, SARS-CoV-2, rheumatoid arthritis

## Abstract

Thrombocytopenia is defined as a platelet count below 150,000/mm^3^ for adults. There is still controversy about whether individuals with platelet counts of 100,000/mm^3^ to 150,000/mm^3^ should be classified as having genuine thrombocytopenia or borderline thrombocytopenia. Thrombocytopenia is considered mild when the platelet count is between 70,000 and 150,000/mm^3^ and severe if the count is less than 20,000/mm^3^. Thrombocytopenia in rheumatoid arthritis is a rare complication, with an incidence estimated between 3 and 10%. The main etiological aspects include drug-induced thrombocytopenia and immune thrombocytopenic purpura. The most common hematological abnormalities in SARS-CoV-2 infection are lymphopenia and thrombocytopenia. It has been observed that the severity of thrombocytopenia correlates with the severity of the infection, being a poor prognosis indicator and a risk factor for mortality. COVID-19 can stimulate the immune system to destroy platelets by increasing the production of autoantibodies and immune complexes. Autoimmunity induced by viral infections can be related to molecular mimicry, cryptic antigen expression and also spreading of the epitope. During the COVID-19 pandemic, it is of great importance to include the SARS-CoV-2 infection in differential diagnoses, due to the increased variability in forms of presentation of this pathology. In this review, our aim is to present one of the most recently discovered causes of thrombocytopenia, which is the SARS-CoV-2 infection and the therapeutic challenges it poses in association with an autoimmune disease such as rheumatoid arthritis.

## 1. Introduction

Megakaryocytes produce platelets, while thrombopoietin controls their formation and maturation in bone marrow. Platelets are involved in thrombosis, wound healing, inflammation, the immune system and cancer biology. Platelet counts typically range from 150,000 to 450,000/mm^3^. Thrombocytopenia is defined by a platelet count below 150,000/mm^3^ for adults. There is still controversy about whether individuals with platelet counts of 100,000/mm^3^ to 150,000/mm^3^ should be classified as having genuine thrombocytopenia or borderline thrombocytopenia [1,2]. Thrombocytopenia is defined as mild when the platelet count is between 70,000 and 150,000/mm^3^ and severe if the count is less than 20,000/mm^3^ [3].

The extrinsic clotting route includes platelets, among other factors. The undamaged vascular endothelium is typically smooth and secretes nitric oxide, whose role is to prevent platelets from adhering. Thrombocytes adhere to the vascular endothelium when damage to the lining occurs. The platelets release nucleotides, adhesive proteins, growth factors and pro-coagulants, which cause thrombocytes to aggregate and blood clots to form, but also can attract other platelets to the site of injury. The fibrin mesh formed from the intrinsic clotting cascade strengthens the formed platelet plug [4,5].

Bleeding induced by poor primary hemostasis and platelet plug formation is a prominent clinical consequence of thrombocytopenia. Thrombocytopenia can occur from decreased bone marrow production, increased destruction of platelets and sequestration. Common causes of thrombocytopenia include primary immune thrombocytopenia, drug-induced immune and non-immune thrombocytopenia, infections, autoimmune disorders, myelodysplasia, malignancy or inherited thrombocytopenia [6,7].

In this review, our aim is to present one of the most recently discovered causes of thrombocytopenia, which is the severe acute respiratory syndrome coronavirus 2 (SARS-CoV-2) infection, by corroborating the recent literature data on SARS-CoV-2 infection, rheumatoid arthritis (RA) and thrombocytopenia.

## 2. Causes of Thrombocytopenia

### 2.1. Decreased Production

*Viral infections* such as hepatitis B and C viruses, Epstein–Barr, cytomegalovirus (CMV), parvovirus B19, varicella-zoster virus, rubella and mumps, but also human immunodeficiency virus (HIV) and SARS-CoV-2, can cause thrombocytopenia due to decreased bone marrow production of platelets [8].

Bacterial and rickettsial infections such as tuberculosis, malaria and Lyme disease may cause a decrease in platelet count due to destruction mediated by the immune system, splenic sequestration and also a shorter thrombocyte survival period. Tuberculosis may also lead to immune thrombocytopenic purpura (ITP) [9].

Malignancies include myeloid syndromes such as myelodysplastic and myeloproliferative disorders, lymphoid malignancies such as leukemia, non-Hodgkin’s lymphoma, multiple myeloma and Waldenström macroglobulinemia, but also prostate or breast cancer [10].

Bone marrow suppression due to radio and chemotherapy may also be a cause of thrombocytopenia [11].

Liver disease can lead to a decrease in platelet count in the case of alcoholic cirrhosis, alcohol abuse, nonalcoholic fatty liver disease, drug-induced liver disease and infections, but also metabolic hepatitis [12].

### 2.2. Sequestration

In diseases such as sarcoidosis, some lymphomas, Gaucher’s disease and Felty syndrome, the platelet count may seem low due to splenic sequestration, although the total numbers are normal or increased. This process is due to the fact that reticular cells may interpret thrombocytes as being foreign and remove them from the circulation [13].

### 2.3. Increased Destruction

Autoimmune disorders such as immune thrombocytopenic purpura (ITP), systemic lupus erythematosus, sarcoidosis, rheumatoid arthritis, mixed connective tissue disease or Graves’ disease are causes of thrombocytopenia. Antiphospholipid syndrome associated with pulmonary emboli in the presence of thrombocytopenia is related to an immune-mediated mechanism [14].

Disseminated intravascular coagulation (DIC) is caused by a dysregulation of the clotting mechanisms which is associated with bleeding, but also clotting, in the microcirculation. The causes of DIC range from severe trauma to transplant rejection and malignancies [15].

### 2.4. Drug-Induced Thrombocytopenia

Many drugs have been incriminated in the development of thrombocytopenia, mostly either by immune-mediated destruction or impaired platelet production. Sometimes, the causative agent is difficult to identify. Medications such as chemotherapy in high doses, chloramphenicol, phenylbutazone, heparin, clopidogrel and other immunosuppressive agents have been cited as inducing thrombocytopenia [16].

### 2.5. Pregnancy

Gestational thrombocytopenia is caused by the expanded plasma volume and is usually benign. Preeclampsia, eclampsia and HELLP syndrome have also been associated with low platelet counts [17].

## 3. Clinical Experience—Case Report

A 70-year-old man with a 10-year history of anti-citrullinated protein antibodies (ACPA) positive RA, currently treated with Leflunomide 10 mg/day and Sulfasalazine 2 g/day, was referred to our clinic in April 2021 due to pain and swelling of the small joints of the hands and prolonged morning stiffness. The patient’s most recent medical history included a right middle cerebral artery (MCA) stroke which occurred 2 days after the 1st dose of Pfizer/BioNTech Coronavirus Disease 2019 (COVID-19) mRNA vaccine in January 2021 and an asymptomatic SARS-CoV-2 infection in March 2021. Regarding the stroke, the patient was investigated in the Neurology Department for possible risk factors such as atherosclerosis (since RA is a well-known risk factor for an accelerated atherosclerotic process), but no atherosclerotic plates were found on the carotid ultrasound, and no cardiac arrhythmias, arterial hypertension, dyslipidemia or clotting disorders were discovered. He was treated during hospitalization with Low Molecular Weight Heparin (LMWH) at therapeutic dosage and switched to double antiaggregation (Clopidogrel and low dose aspirin (LDA)) at discharge. The platelet count during the admission was within normal range (220,000/mm^3^). The patient followed the usual vaccine schedule after the stroke.

From the prescription history of the rheumatic disease, we mention that the patient received multiple conventional synthetic disease modifying anti-rheumatic drugs (DMARDs) with good tolerance and without side effects, as follows: Methotrexate (MTX) between 2011 and 2018 at a dose of 15 mg per week, Sulfasalazine (SSZ) 2 g per day from 2011 to present and Leflunomide (LFN) 10 mg per day from 2018 to present.

At the first evaluation in our clinic, physical examination and laboratory tests showed high disease activity with a DAS28-CRP score of 5.39 and mild thrombocytopenia of 97,000/mm^3^.

The abdominal ultrasound was unremarkable. We considered the thrombocytopenia to be mild and transient and decided, due to high disease activity, to increase the LFN dosage to 20 mg/day (therapeutic dosage), in addition to SSZ and a moderate dose of Prednisone (10 mg/day) with gradual tapering.

A month later, due to the significant decrease in platelet count up to 46,000/mm^3^ we decided to stop immunosuppressive therapy (LFN and SSZ) and refer the patient to the hematology clinic, where he was thoroughly investigated. The venous blood platelet count was 19,000/mm^3^, while the mean capillary blood count showed approximately 100,000/mm^3^. The bone marrow biopsy and platelet antibodies profile revealed no abnormalities, whereas deficient folic acid and B12 levels were present. Therefore, the low levels of platelets were considered in this instance to be related to a folate and B12 deficiency. The patient was prescribed vitamin B12 and folic acid. Nevertheless, an immune-related thrombocytopenia triggered by the SARS-CoV-2 infection could not be ruled out.

On 27 May, 3 weeks after stopping the DMARDs, the patient returned to the rheumatology clinic with numerous painful and swollen joints, musculoskeletal ultrasound and laboratory tests showing increased activity of RA. The platelet count was still low (42,000/mm^3^), but the mean capillary blood count showed 100,000/mm^3^. Due to high disease activity (DAS28-CRP = 7.29, C-Reactive Protein (CRP) = 141.49 mg/L), after excluding an infectious etiology due to normal white blood cell count and neutrophils and normal procalcitonin levels, we decided to initiate MTX at a dosage of 10 mg per week with methylprednisolone 16 mg daily with close hematological surveillance.

**Figure 1:**
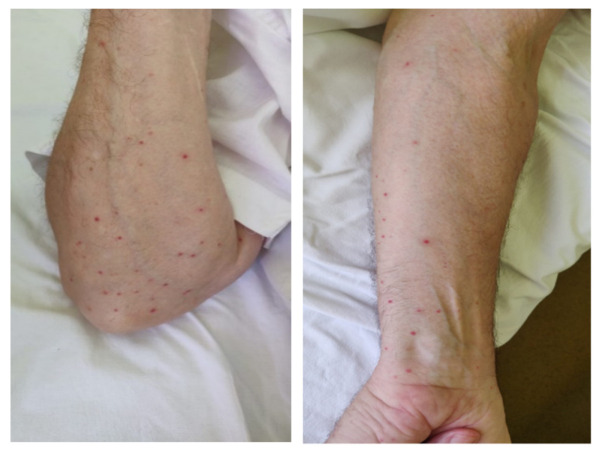
Petechial rash of the limbs.

On 17 June, the patient presented petechiae located on the limbs, with no signs of active bleeding and partial relief of joint symptoms (Figure 1). The disease activity was persistently high (DAS28-CRP = 5.59) with important biologic inflammatory syndrome (CRP = 23.14 mg/L). The laboratory tests showed severe thrombocytopenia (6000/mm^3^) with normal coagulation tests. We also determined the antiphospholipid and antiplatelet antibodies which were negative.

As a result of the interdisciplinary evaluation, the hematologist recommended Dexamethasone 32 mg/day intravenously for 5 days and 2-unit platelet transfusions, leading to a rapid increase in platelet count. After 5 days, the platelet count increased to 98,000/mm^3,^ and the patient was referred to the Hematology Department for further investigations. After excluding myelodysplastic syndromes (normal bone marrow biopsy), considering the negative platelet antibody profile and worsening thrombocytopenia despite supplementation with vitamin B12 and folic acid, we established the diagnosis of COVID-19-associated thrombocytopenia enhanced by the immunosuppressive therapy (MTX).

Due to worsening thrombocytopenia with steroid tapering and high RA activity, we decided to administer a therapy that would target efficiently both the hematological and rheumatic disease, since conventional synthetic and biological DMARDs can induce thrombocytopenia. We concluded that the most appropriate therapeutic approach is monoclonal anti-CD20 antibody therapy with Rituximab, effective in the treatment of both RA and immune thrombocytopenia.

On 2 July, he returned to the Rheumatology Clinic for the first infusion of Rituximab. The patient had a platelet count of 22,000/mm^3^ and moderate activity of RA (DAS28-CRP = 4.92), under treatment with a moderate dose of steroids. At re-admittance, 2 weeks later, for the second Rituximab infusion, platelet levels had doubled, reaching 56,000/mm^3^, with slight improvement in disease activity (DAS28-CRP = 4.38).

**Figure 2:**
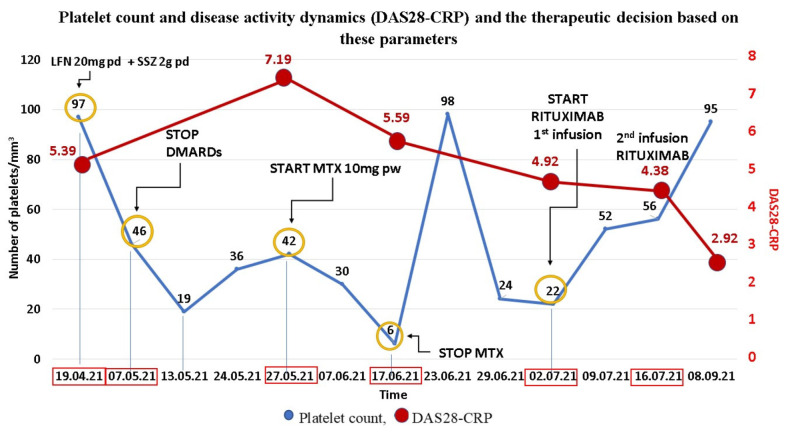
Platelet count and disease activity dynamics (DAS28-CRP) and the therapeutic decision based on these parameters.

On 8 September, 2 months after initiating biological therapy, he was in good clinical condition, with a single painful joint and no morning stiffness. According to EULAR guidelines, the patient achieved “treat to target” strategy, having low disease activity (DAS28-CRP = 2.92, ΔDAS = 1.46). The platelet count increased to 95,000/mm^3^ (Figure 2).

Stroke and thrombocytopenia, during the pandemic, have to be carefully analyzed, because SARS-CoV-2 vaccine and infection could be involved in their etiology. Regarding the stroke, we excluded other possible causes of hyperviscosity, as well as arterial hypertension and diabetes mellitus.

COVID-19 infection can cause multiple hematological changes, one of which is thrombocytopenia, which can be severe and life-threatening.

Our patient was previously vaccinated with an mRNA vaccine and had an asymptomatic infection with the SARS-CoV-2 virus, showing a reactivation of RA in addition to continuously aggravating thrombocytopenia, despite treatment with glucocorticoids. In collaboration with the hematologist, we investigated the cause of low platelet count. A myelodysplastic syndrome was considered improbable (normal bone marrow biopsy) and the platelet-associated immunoglobulin G (PA-IgG), was unremarkable; thus, we suspected that thrombocytopenia was secondary to vitamin B and folic acid deficiency. The platelet count continued to decrease, even though the patient received vitamin B and folic acid supplements in combination with glucocorticoids. After excluding other causes, we concluded that the patient had COVID-19-associated thrombocytopenia, aggravated by the DMARDs administered. Because the patient had sustained high activity of RA and worsening thrombocytopenia, we had to take immediate and effective therapeutic measures. To address both entities, after consulting the latest rheumatology and hematology guidelines [18,19] and considering the non-response to corticosteroid therapy, we decided to start treatment with monoclonal anti-CD20 antibody, Rituximab. The patient responded very well to biological therapy, showing an important decrease in RA activity with a significant and sustained improvement in platelet count two months after the initiation of the biological therapy.

We did not take into account the implication of a SARS-CoV-2 vaccine, but we considered the SARS-CoV-2 infection a trigger factor for this relapse due to the closeness to the reactivation of RA. Regarding this issue, a study in Switzerland demonstrated that during the pandemic, the risk of developing a flare in patients with RA, psoriatic arthritis and ankylosing spondylitis was 15% higher than in the pre-COVID period [20].

The particular aspects of our case were that the SARS-CoV-2 infection was asymptomatic, the duration from COVID-19 illness to the first detection of thrombocytopenia was almost 36 days, the main clinical manifestation was petechiae, the response to corticotherapy was limited, the patient needed platelet transfusions, the antiplatelet autoantibodies were negative and the DMARDs used for RA led to an immediate drop in platelet count. In this very complex situation, Rituximab targeted both entities, a good outcome regarding RA activity and immune thrombocytopenia being rapidly noticed.

## 4. Rheumatoid Arthritis-Related Thrombocytopenia

Rheumatoid arthritis (RA) is a chronic inflammatory autoimmune disease that primarily affects the small joints and may present hematological manifestations. Thrombocytopenia in RA is a rare complication, with an incidence estimated as high as 3–10% in some reports. However, other authors state that the incidence is between 4.1 and 4.7%, while in other recent literature reports it was discovered in less than 1% of patients with RA [21,22,23,24,25]. The main etiological aspects include drug-induced thrombocytopenia and ITP [26].

The most frequent cause of thrombocytopenia among patients with RA is drug-induced. Most disease-modifying anti-rheumatic drugs (DMARDs), either conventional synthetic (Methotrexate, Leflunomide, Sulfasalazine), biological therapy (TNF alpha inhibitors, IL-6 inhibitors) or targeted synthetic (JAK inhibitors), have thrombocytopenia among the reported adverse events [21,27]. One of the mechanisms involved is direct drug toxicity which suppresses bone marrow, leading to decreased platelet production. The drug-induced thrombocytopenia can also be caused by drug-dependent antibodies that bind to the platelet membrane glycoproteins, mainly GP IIb/IIIa or Ib/IX, leading to impaired platelet production and reduced lifespan of the thrombocytes, the majority being cleared in the spleen [28].

Although TNF-inhibitors may cause cytopenias, the specific induction mechanism is unknown. Because TNF regulates pro-inflammatory cytokines including interleukin (IL)-1, IL-6, IL-8 and granulocyte-macrophage colony-stimulating factor (GM-CSF), it has the potential to impede stem-cell differentiation and cause bone marrow failure. A lupus-like condition or direct marrow damage are two of the most probable explanations [29].

Although rare, there are cases reported in the literature of the association between RA and ITP. ITP is characterized by a low platelet count in the absence of other causes of thrombocytopenia and associates increased megakaryocytes in bone marrow that release immature platelets into circulation. The autoantibodies bind to the surface antigens of the thrombocyte membrane and cause their peripheral destruction by phagocytosis in the spleen, ITP being considered a form of hypersplenism. Splenomegaly can also be seen in ITP. Several studies have demonstrated that the association RA-ITP may be more resistant to glucocorticoids than ITP alone [30].

Another potential cause of thrombocytopenia is folic acid or vitamin B12 deficiency, which in fact has become less common due to the recommendation of folic acid supplementation when using immunosuppressive therapy.

A desirable course of action in the treatment of thrombocytopenia is the cessation of the DMARD and the administration of glucocorticoids in tapering doses [31].

## 5. SARS-CoV-2 Infection-Induced Thrombocytopenia

The most common hematological abnormalities in SARS-CoV-2 infection are lymphopenia, cited in literature in up to 83.2% of patients and thrombocytopenia in 5–21% of cases [32]. Guan et al. reported an incidence of 36.2% for thrombocytopenia in a cohort of 1099 patients from China [33,34]. It has been observed that the severity of thrombocytopenia correlates with the severity of the infection, being a poor prognosis indicator and a risk factor for mortality [32,35].

**Figure 3:**
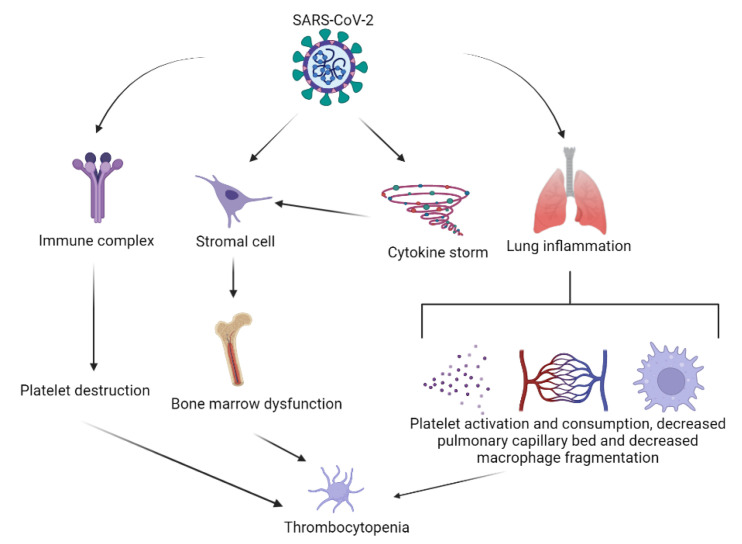
Mechanisms of thrombocytopenia in COVID-19.

The potential mechanisms of thrombocytopenia in COVID-19 involve:

- Platelet activation and subsequent clearance by reticuloendothelial system;
- Platelet clearance due to increased endothelial damage;
- Platelet autoantibody formation, with subsequent platelet clearance;
- Splenic/hepatic sequestration;
- Marrow/megakaryocyte suppression;
- Antiretroviral drugs [36] (Figure 3).

SARS-CoV-2 has the ability to directly infect bone marrow and inhibit hematopoiesis, leading to platelet synthesis. Similarly, it inhibits hematopoiesis in bone marrow through certain receptors to cause decreased primary platelet formation which leads to thrombocytopenia. The nucleotide homology between SARS-CoV-2 and human SARS-CoV (HCoV-229E) is 82%. Because SARS-CoV and HCoV-229E exhibit equivalent antigen features, it is possible that the antigens of SARS-CoV-2 and HCoV-229E are related. Human aminopeptidase N (CD13) is a metalloprotease found on the cell surfaces of epithelial cells in the colon, kidneys and lungs, and is an HCoV-229E receptor [37,38]. CD13 is a granulocyte and monocyte marker found in epithelial cells of the respiratory tract, smooth muscle cells, fibroblasts, kidney and small intestine epithelial cells, activated endothelial cells, lymphocytes and platelets. Through CD13 receptors, HCoV-229E reaches bone marrow cells and platelets, causing growth inhibition and apoptosis in bone marrow, resulting in abnormal hematopoiesis and thrombocytopenia. SARS-CoV-2 infection causes thrombocytopenia comparable to that induced by SARS-CoV and HCoV-229E infection [39,40]. SARS-CoV-2, based on this phenomenon, is thought to inhibit hematopoiesis in bone marrow via certain receptors, resulting in decreased primary platelet formation and thrombocytopenia [28].

Another mechanism of decreased platelet production is secondary hemophagocytic lymphohistiocytosis (sHLH) [41]. sHLH is caused by excessive mononuclear macrophage expansion and activation, which results in the production of a high number of inflammatory cytokines and the consumption of a significant number of blood cells. The basic features of this reactive disease include persistent fever, hyperferremia, cytopenia and lung involvement, having a rapid response with a high mortality rate. Elevated ferritin was revealed to be one of the predictors of fatality. In the case of SARS-CoV-2 infections, the T lymphocytes are overactivated and produce GM-CSF and IL-6 [42,43]. GM-CSF stimulate inflammatory mononuclear macrophages to produce increasing amounts of IL-6, resulting in an inflammatory storm and immunological damage to the lungs and other organs. Furthermore, investigations have revealed that cytokine spectrums comparable to sHLH are linked to the severity of COVID-19 illness. The hematopoietic progenitor cells in bone marrow of patients with pneumonia caused by SARS-CoV-2 were destroyed after the cytokine storm, while the primary production of platelets declined, and at the same time, too many blood cells were consumed, resulting in a decrease in peripheral blood platelet count [44,45].

Damage to the pulmonary endothelial cells can activate and/or aggregate platelets in the lungs leading to microthrombi formation and consequently thrombocyte consumption. The majority of patients infected with SARS-CoV-2 who have thrombocytopenia, also have elevated D-dimer levels, in addition to impaired coagulation time. This speaks to the hypothesis that there is low intravascular coagulation [46].

COVID-19 can stimulate the immune system to destroy platelets by increasing the production of autoantibodies and immune complexes. Autoimmunity induced by viral infections can be related to molecular mimicry, cryptic antigen expression and also spreading of the epitope [47]. Molecular mimicry is well recognized in the case of HIV, hepatitis C or varicella zoster viruses, leading to the formation of cross-reactive antibodies to glycoproteins (GP) on the surface of the platelets. Antibodies against GP IIb/IIIa, GP Ib/IX or GP V have been found in a number of instances, while sequence homology between SARS-CoV-2 and thrombocyte components has yet to be revealed. Antiplatelet antibodies and immune complexes cover platelets, causing them to be cleared by the reticuloendothelial system [29,44]. Viruses can cause the expression of cryptic antigens by infecting cells directly. Angiotensin-converting enzyme 2 (ACE 2) receptors are not found on platelets. Recent research, however, found that platelets may utilize SARS-CoV-2 mRNA without ACE 2 [48].

Lower levels of regulatory T cells have been discovered in immune dysregulated COVID-19 patients. More than one-third of immunogenic proteins in SARS-CoV-2 have homologous proteins, part of the immune adaptive system in humans. In these cases, the development of immune thrombocytopenia is related to MHC class I and II antigen presentation and also PD-1 signaling due to an abnormal immune response to the homologous proteins [49,50,51,52].

Guan et al. analyzed 1099 COVID-19 patients hospitalized in China at the beginning of the epidemic and found out that 36.2% of them had thrombocytopenia on admission. They also showed an association between the severity of thrombocytopenia and SARS-CoV-2 infection. A meta-analysis of nine studies supports these findings [32].

A systematic review of 45 cases of immune thrombocytopenia secondary to COVID-19 concluded that the majority of the patients were elderly and had a moderate-to-severe type of infection. Furthermore, 31% of the patients showed no signs of active bleeding, while life-threatening bleeding was unusual, petechia being described as a clinical manifestation in half of the cases. The duration between COVID-19 illness and the diagnosis of immune thrombocytopenia was a median of 13 days (minimum 7 and maximum 21 days). Most of the patients received glucocorticoids and intravenous immunoglobulin (IV IG) with rapid improvement. Thrombopoietin receptor agonists were used as second-line agents due to the potential risk of hepatotoxicity and thrombotic complications. Some of the patients with signs of active bleeding also received platelet transfusions. All of the patients showed good initial response to steroids and IV IG, except for one case which needed Rituximab as saving therapy, due to the coexistence of Evans Syndrome (immune hemolysis) [46].

## 6. SARS-CoV-2 mRNA Vaccine-Related Thrombosis and Thrombocytopenia

Studies have shown an increased risk of venous thromboembolism, arterial thrombosis, cerebral venous sinus thrombosis, ischemic stroke and myocardial infraction after COVID-19 vaccine, but the risk is considered lower than after SARS-CoV-2 infection [53].

Regarding the COVID-19 vaccination, there is increased suspicion related to the implication of the vaccines in thrombotic events. In relation to these issues, Al-Mayhani et al. described three cases of ischemic stroke (thrombosis of large cerebral vessels) associated with COVID-19 vaccination. The particularity of these cases consists in the association of thrombocytopenia with the immune-mediated coagulopathy [54].

A nationwide study conducted in France indicated that cardiovascular risk is not increased in elderly patients after Pfizer’s COVID-19 vaccine: the relative incidence for ischemic stroke is 0.90 after the first dose (95% CI, 0.84–0.98) and 0.92 (95% CI, 0.84–1.02) after the second dose [55].

There are cases of thrombocytopenia following mRNA COVID-19 vaccines reported in the literature that represent in fact adverse events as defined by the Vaccine Adverse Events Reporting System [56,57]. None of the 15 cases of thrombocytopenia reported after Pfizer-BioNTech COVID-19 vaccine did not associate ITP before vaccination, only two of the cases having a history of chronic diseases, one with Crohn’s disease and another with type 1 diabetes mellitus [56]. In this report, Rituximab was used to control severe thrombocytopenia in two cases, both exposed to the Pfizer-BioNTech COVID-19 vaccine, neither to the Moderna COVID-19 vaccine [57].

In September 2021, a Japanese group reported three cases of post-vaccine severe thrombocytopenia among elderly patients with seropositive (ACPA, rheumatoid factor (RF)) RA. One of the patients was previously diagnosed with ITP secondary to Sarilumab and was controlled by intravenous immunoglobulin and prednisolone administration, having a normal platelet count at the time of vaccination. After the vaccine, he developed a flare of ITP, efficiently treated with higher dosage of corticosteroids in association with Tacrolimus. The other two patients experienced a longer duration of RA and were treated initially with Methotrexate and then switched to Golimumab. They developed marked thrombocytopenia 4 days after the first dose of BNT162b2 vaccine. It is important to mention that both of them are associated with chronic kidney disease (CKD) under treatment with epoetin beta pegol and also one of them had Hashimoto’s disease. Regarding the platelet-associated immunoglobulin G (PA-IgG), two of the three patients developed high titers of PA-IgG, being negative for ANA and anti-dsDNA antibody. Furthermore, two of the patients were hospitalized for severe bleeding complications (subarachnoid and subcortical hemorrhage) [58]. After this detailed description of the severe thrombocytopenia diagnosed in patients with RA in the first week after the first dose of mRNA vaccines, we could exclude this etiology for the severe thrombocytopenia in our case.

Rarely, patients with RA vaccinated with mRNA vaccines could develop a manageable and reversible flare of their disease, especially during the first month post vaccination. After the complete vaccination schedule, the risk of flare was limited; thus, the patients should have no fear in receiving either mRNA vaccines [59].

In conclusion, evidence is contrasting in demonstrating an increased risk of thromboembolic events, either arterial or venous or thrombocytopenia, after COVID-19 mRNA vaccine and needs to be balanced against the risk of contracting the virus itself.

## 7. Rheumatoid Arthritis and SARS-CoV-2

Despite the numerous variations between SARS-CoV-2 infection and RA in terms of origin, epidemiology, clinical characteristics, organ involvement and prognosis, the pathophysiology and risk factors linked with these diseases appear to be somewhat comparable [60].

In terms of risk factors, advanced age appears to increase the chance of developing RA. This might be attributed to substantial changes in lymphocyte populations and morphologies, which could lead to an increase in self-tissue antigen reactivity. Similarly, being over 65 years old may raise the overall chances of contracting COVID-19. Smoking is another known risk factor, with smokers developing seropositive, more erosive RA with extraarticular manifestations. Similarly, smoking raises the risk and severity of COVID-19, which is linked to lung damage and decreased lung capacity [61].

Infections provide a serious risk to people with inflammatory arthritis, since they can trigger disease flares. Furthermore, RA patients frequently have comorbidities such as diabetes, cardiovascular disease and pulmonary illness, all of which enhance the risk of viral infections. This higher risk of infection is linked to several of the same risk variables identified in COVID-19 [62].

Vasculitis is another manifestation associated with RA and COVID-19 infection. One of the most common complications associated with RA is systemic rheumatoid vasculitis. It is marked by inflammation of the mid-size arteries and capillaries, which can progress to deep cutaneous ulcers, gangrene, and neuropathy, all of which are linked to poor outcomes and mortality. Some case studies have recently acknowledged the existence of vasculitis in SARS-CoV-2 patients [63]. 

The cytokine imbalance seen in COVID-19 infection is strikingly comparable to that seen in inflammatory rheumatic illnesses. This includes the pro-inflammatory cytokines such as interleukin (IL)-1, IL-6 and tumor necrosis factor (TNF)-α, as well as the inflammatory chemokines CCL-2 and CXCL-10, which have previously been seen in other coronavirus infections such as Middle East respiratory syndrome (MERS). Individuals with severe COVID-19 had a lower number of CD4+ and CD8+ T lymphocytes, as well as increased levels of TNF-, IL-1 and IL-6 in their plasma. It is worth noting that IL-6 is one of the most important inflammatory mediators in COVID-19, with its levels being linked to the viral load. Furthermore, genetic host traits such as IL-6 gene polymorphisms, which might contribute to SARS-CoV-2 susceptibility and RA, are similar in COVID-19 and RA [64,65].

The mechanism-based similarity between COVID-19 and RA is based on the angiotensin converting enzyme (ACE)-dependent pathway and the macrophage-dependent pathway.

### 7.1. The ACE-Dependent Pathway

SARS-CoV-2 may use ACE2 as a possible cellular target for entry into target cells. Although ACE2 binding is required for SARS-CoV-2 entrance into host cells, recent data suggest that heparan sulfate plays a vital role in promoting their connection and therefore potentiating SARS-CoV-2 cell entry and infection. ACE2 as an enzyme, on the other hand, protects against SARS-CoV-2 infection. SARS-CoV-2’s S-protein binds to ACE2, causing ACE2 expression to be suppressed and COVID-19 pathogenesis to be promoted. Inhibition of ACE2/angiotensin-1–7 increases the Raf/mitogen-activated protein kinase (MAPK) pathway, which shares pathogenic signaling in COVID-19 and RA [66,67,68].

ACE activation enhances the production of angiotensin II, which may have a role in COVID-19 and RA pathology. Angiotensin II is known to stimulate inflammatory responses and vascular permeability in an inflammatory environment by increasing the synthesis of prostaglandins and vascular endothelial growth factor (VEGF). These inflammatory mediators also increase the activation of nuclear factor kappa-light-chain-enhancer of activated B cells (NF-B), which enhances inflammatory responses and promotes inflammatory cell infiltration into injured tissues. Angiotensin II can also promote lymphocyte proliferation and activation, as well as the production of free radicals in leucocytes. ACE inhibitors and angiotensin receptor blockers have been shown to be effective in COVID-19, delaying SARS-CoV-2 binding by activating ACE2 and boosting angiotensin-1–7 availability [69,70]. It is possible to say that COVID-19 and RA have the same immunopathogenic mechanism driven by abnormal ACE/ACE2 activity [48].

### 7.2. The Macrophage-Dependent Pathway

The macrophages found in the bronchial and synovial tissues are diverse. Alveolar macrophages that produce fatty-acid-binding protein 4 (FABP4) are aiding sustained gas exchange and compliance in healthy lungs during the SARS-CoV-2 outbreak. The number of FABP4-expressing alveolar macrophages decreases dramatically after infection. As a result, gas exchange is impeded [71]. COVID-19 pathogenesis most likely implies the involvement of macrophages. CD8+ T cells mediate the powerful adaptive immunological response. Their precise pathogenic function, however, has yet to be determined. In RA patients, synovial tissue also has unique macrophage subsets when compared to healthy persons. When compared to healthy joints, synovial tissue from RA patients contains two distinct types of macrophage clusters that have been linked to RA pathogenesis by producing pro-inflammatory mediators such as IL-1, IL-6, TNF-α, matrix metalloproteinases (MMPs) and chemokines, as well as inducing pathogenesis in adjacent stromal tissue. Some macrophages in COVID-19 patients’ bronchoalveolar lavage fluids have a transcriptional resemblance to pathogenic macrophage clusters in RA patients’ synovial tissue. The macrophages in COVID-19 patients’ alveolar tissue are similar to the synovial macrophages in RA patients. As a result, both SARS-CoV-2 infection and RA have a comparable immunopathogenic mechanism driven by homologous macrophage clusters [65,72,73].

In conclusion, COVID-19 and RA have immune-inflammatory aspects of disease pathogenesis that are triggered by comparable mechanisms.

## 8. Management of RA and Thrombocytopenia in COVID-19 Patients

Clinical management of RA is a difficult task in the current COVID-19 environment. The American College of Rheumatology (ACR) and the European Alliance of Associations for Rheumatology (EULAR) proposed many recommendations for the use of RA medication in the COVID-19 pandemic among possible therapy alternatives. Even in COVID-19-positive patients, glucocorticoids are prescribed at the lowest feasible dose, and abrupt cessation is avoided. Unless severe COVID-19 manifestations are present in several organs, non-steroidal anti-inflammatory medications (NSAIDs) might well be maintained. Conventional synthetic DMARDs (csDMARDs) may be continued in the absence of SARS-CoV-2 infection. However, recommendations for drugs such as leflunomide, methotrexate and sulfasalazine should be avoided in suspected or confirmed COVID-19 cases. In suspected or confirmed patients of COVID-19, all biological DMARDs (bDMARDs) besides IL-6 inhibitors, as well as all targeted synthetic DMARDs (tsDMARDs), should be ceased [74,75].

No clear link has been demonstrated between specific RA medications and the development or consequences of COVID-19, and other reports imply that RA patients are more susceptible to serious infections. Glucocorticoids have a protective function in COVID-19 infection, which is primarily mediated through their immunosuppressive actions to counteract hyperinflammatory states in the late stages of SARS-CoV-2 infection, according to emerging data. Glucocorticoids (10 mg/day) are linked to an increased percentage of COVID-19 hospitalization in patients with rheumatic diseases, according to the CVODI-19 Global Rheumatology Alliance and other reports. High dosages of glucocorticoids have been identified as a possible contributing factors for COVID-19 patients with rheumatic illnesses [76].

According to several findings, using NSAIDs does not cause any major side effects in COVID-19 patients. In situations of severe COVID-19 symptoms, the ACR suggested ceasing NSAIDs. The role of NSAIDs in the treatment of viral infections is still debated. NSAIDs decrease the inflammatory response in SARS-CoV-2 infection, but they also impede the humoral immune response to SARS-CoV-2 infection by reducing the development of protective antibodies, according to recent preclinical studies. Because of their antipyretic principle, the use of NSAIDs in the COVID-19 scenario may cause a delay in the detection of SARS-CoV-2 infection. Treatment with selective COX-2 inhibitors (diclofenac, meloxicam, and celecoxib) was not linked to an increase in COVID-19 severity, however [77,78].

As csDMARDs for the treatment of RA, hydroxychloroquine and chloroquine are routinely utilized. Both of these antimalarial medicines meet the theoretical parameters for COVID-19 effectiveness. As a result, these medications were first included in COVID-19 treatment. However, their impact on COVID-19 management is still debatable. Treatment with chloroquine and hydroxychloroquine has been observed to cause QT prolongation in nearly 10% of COVID-19 patients. In the case of SARS-CoV-2 infection, the ACR has suggested a temporary cessation of hydroxychloroquine and chloroquine for RA patients. Other csDMARDs, such as leflunomide, methotrexate and sulfasalazine, have also been suggested to be temporarily discontinued in RA patients with active SARS-CoV-2 infection [79,80].

In the COVID-19 context, it has been hypothesized that bDMARDs might be effective. However, the impact of bDMARDs on the COVID-19 model is still debatable. Anti-TNF medication has been shown to reduce the severity of the illness and mortality. COVID-19 outcomes were also positively or equivocally affected by anti-IL-6 (tocilizumab) medication. Despite certain medical societies’ recommendations against starting or continuing bDMARDs (including anti-TNF medications) in areas where COVID-19 has been circulating in the population, anti-IL-6 medications have been deemed to be safer. Following COVID-19 exposure, medical groups advised RA patients to temporarily stop using tsDMARDs. Withholding DMARDs for a period of time might increase the risk of disease recurrence and worsening morbidity in RA patients. As a result, treating RA patients who have acquired SARS-CoV-2 infection and are being treated with DMARD medicines for RA will be a difficult challenge for physicians [80,81].

Regarding the management of COVID-19-induced thrombocytopenia, some of the current guidelines state that observation alone should be the elected course of treatment. If medication is necessary, corticosteroids are usually the first line of treatment, with a suggested prednisone dose of 1 mg/kg/day orally for up to 21–28 days, depending on response, followed by careful tapering. Repeated pulses of high-dose dexamethasone 40 mg day for four days reportedly produce more pronounced platelet responses, but no comparable data in favor of dexamethasone exist [82,83].

In addition, with or without steroids, intravenous immunoglobulin or intravenous anti-D (Rho[D] immune globulin) might be utilized as a first therapy. A brief course of high-dose intravenous immunoglobulin (1 g/kg for 1–2 days) or an initial intravenous immunoglobulin dosage of 0.4 g/kg/day for up to 5 days has been proven to be beneficial. After first treatment, the majority of adult patients relapse (or are unresponsive to first-line therapy) and require second-line therapy [84].

Many medicines, including azathioprine, cyclosporine, cyclophosphamide, danazol, dexamethasone, vinca alkaloids, mycophenolate mofetil, rituximab and thrombopoietin-receptor agonists, are additional second-line therapeutic options with established evidence of success. Thrombopoietin receptor agonists can lead to a steady rise in platelet count and also prevent relapse. Other options included eltrombopag, romiplostim and platelet transfusions [84,85].

The anti-CD20 monoclonal antibody rituximab is commonly used in ITP, in a variety of regimens and combinations with other medications, such as dexamethasone. The most typical rituximab treatment is a 375 mg/m^2^ intravenous infusion administered once weekly for four weeks [83,86,87].

Understanding the underlying pathophysiological mechanisms in the development of thrombocytopenia is a key component of optimal therapy.

## 9. Conclusions

During the COVID-19 pandemic, it is of great importance to include the SARS-CoV-2 infection in the differential diagnoses, due to the increased variability in forms of presentation of this pathology. As such, an etiological diagnosis of thrombocytopenia must take into account the new emerging cause, which is COVID-19. Furthermore, the association between SARS-CoV-2 infection and rheumatic diseases requires a complex diagnostic algorithm as well as customized management, especially in the case of hematological manifestations. Thus, the management and treatment of RA patients in a COVID-19 setting poses a great challenge to clinicians.

## Data Availability

Not applicable.
